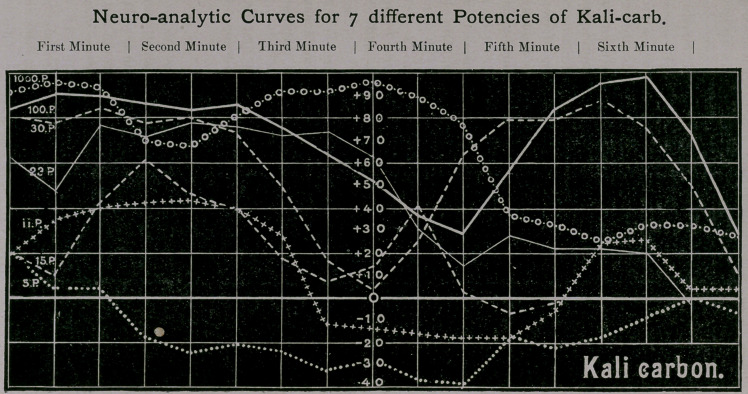# Potentiation Physiologically Proven

**Published:** 1892-08

**Authors:** Gustav Jaeger

**Affiliations:** Stuttgart, Germany


### POTENTIATION PHYSIOLOGICALLY PROVEN.

By Prof. Dr. Gustav Jaeger, Stuttgart, Germany.

[Translated from the Leipzig *Allgem, Homœop. Zeitung*, Vol. 124, No 11, by B. Fincke, M. D.]

Continued from page 239.

*Motto:* All things are poison and nothing is without poison, but the doses alone make it that a thing is no poison. (Paracelsus in his third defense, Barth ed. II, p. 170.)

### II. Introduction.

Hahnemann and before him A. v. Haller, for the proving of medicines, laid down the postulate that in the *first* place it must be made upon the *healthy*. This proposition has also been followed by all the provers in our camp (only in regard to Kochine some enthusiasts were carried away by allopathy and entered immediately on proving it upon the sick). Their proving, however, hitherto—as far as I know—has occupied itself with only one of the two fundamental laws of Homœopathy, viz., with the law of *Simility* while the law of *Potentiation* has been left in the lurch. This unequal treatment of the two principles had also here the following result: (*a*) whilst the practical physician for the qualitative selection of the remedy, disposes over but too large a number of remedies, and all homœopaths are uniform in their acceptation of the *law of Simility*—we pass over the difference of opinion regarding *ison* and *homoion*—the literature regarding the quantitive selection shows us a great difference of opinion.

(*b*) Yea, still more since potentiation, especially the high potencies, form the great stumbling-block on the allopathic side—there are homœopaths who do not hesitate to part with this fundamental law of Homœopathy—no *phraseology* and no one-sided experiments upon the sick can change this state of things, but only new facts which must be obtained by *experiments* upon the *healthy*.

I, from my standpoint, cannot at all comprehend how people can quarrel about such a matter. The only correct thing to do is to take the matter in hand and try it, and try it *upon one's self.*

I have done so, and thereby found—as the reader will see from the following—that any one can, just as Hahnemann did, soon settle the matter, without any other artificial means than perhaps a common watch, but still easier of course, if one does not confine himself to the observation of the internal phenomena, but calls to aid the method which I have called *Neural Analysis.*

Since I have repeatedly published the method exactly and extensively, especially lately in my writing *Action of Matter in Living Beings* (Albert Gunther, Leipzig, 1892), I presume that the readers know all about this method, and beg leave to make a preliminary remark.

Since an exact science exists, the rule in investigations of any kind is adopted if possible to apply a method which gives *numbers.* Already the popular opinion says: "numbers prove" and *Kant* says: "Every single branch of natural science contains only so much true science as it *contains mathematics.*"

Such a method is *Neural Analysis.* It has been tested by me in an uninterrupted practice of almost ten years, and the reader will be enabled by the description of the results of my experiments to form, to a certain degree, his own judgment about this method, but a perfect one indeed only after he has tried it himself. It is a great error to imagine that it would be enough to have dabbled a few times with the chronoscope.

1. Clumsy people, and those with a heavy hand, can learn the Neural Analysis just as little as the use of the microscope and making preparations; this needs a fine touch or a coarser instrument than any chronoscope.

2. *Torpid* people who react upon nothing, who enjoy an ordinary throat-scourer as much as a Rhine wine, of course, also attain nothing. Such people do not come into consideration, because they also as a rule do not fall sick. Upon twenty sick people comes, perhaps, a torpid one, and with him the physician has lots of trouble, because he does not react upon the medicine. Generally the physician has people under treatment who by the fact that they have become sick, prove that they are more "sensitive" natures.

3. A condition which can only be learned by continual exercise and especially numerous experiences to the establishment of the sufficient *spiritual repose*, *passivity*, and *objectivity*.

4. A last main point is that one learns to govern and regulate his physical disposition, which requires invention and again experience.

Finally the following remark: The question of *potentiation* has a *general* and a *special* side, the last in so far as the various medicinal substances in regard to potentiation have not the same relation. I shall treat *both* sides successively, the *general* at first. For this the investigation of a single medicine-substance is sufficient according to its various degrees of attenuation. My selection fell upon *Kali-carbonicum*, just as of course any other substance could have been used for this first fundamental investigation. The *special* or *comparative* side of the question can be only dispensed with by taking care to prove thoroughly a sufficiently large number of medicine-substances in systematic order. This, of course, cannot be done at once and requires several years. Until now I have proved 6 Kali salts, 7 Natrum salts, 4 Ammonia salts.

### III. Neural Analysis of the Potencies of Kali-carbonicum.

### (*a.*) *The Experimentation.*

1. I obtained from the pharmacy the third alcoholic potency. The transference of the substance upon the higher potencies was effected through a series of *equal* vials in order to exclude as much as possible the interference of foreign influences, and was effected by my assistant in another room in order to avoid mixtures of the substance with the air of my room. The table shows which potencies have been measured.

2. Up to the 30th potency the *decimal* scale has been used; from this the *centesimal*, so that one centesimal degree was reckoned equal to two decimal degrees. It will be seen later that this is permissible.

3. Up to the 100th potency alcohol was used, after that up to the 1,000th distilled water, but at the close twice alcohol.

4. For every measurement about two grammes of distilled water, into which one drop of the alcoholic potency was dropped, were swallowed.

5. The decade numbers I dictated to my assistant, who by means of a watch gave me signs at the beginning and the end of the measurement.

6. Each time three measurements were made:

*a.* The *rest*-number formed from four decades, therefore 40 single acts.

*b.* The *water*-number. The same glass from which afterward the mixture of water and medicine should be drank contained a filling only with water. This I drank at a given sign, and measured continually decades till the passage of a minute was signalized. The water-number is the means of all decades obtained, generally 10.

*c.* The *medicine.* Immediately after obtaining the water-number I filled the glass again with water and dropped the drop of medicine in. Upon the sign that a minute was passed I drank the mixture rapidly and commenced the measurement and dictation of the decades. How long is to be seen from the table. The end of every minute was signalized to me.

7. The measurement, as is the general rule for Neural Analysis, took place between 10 and 12 A. M., in a room inaccessible to the exhalations of the kitchen. On January 19th, 7 potencies were proved in this time, on the 20th just as many, on the 21st 2, on the 25th 3 potencies. Before beginning a new measurement it was determined by formation of a rest-number whether the nerve-time had returned to its old standard, and only then when the old stand was reached the operation was continued, otherwise I waited.*

---

* This is not in contradiction with what in No. 1 was said on the inadmissibility of rapidly succeeding measurements of various potencies of the *same* substance. It is *admissible* to measure *approximate* potencies in succession as here was done, but inadmissible when the degrees are *very* different. On this subject exact statements will be made as soon as the practical question has been solved. For the present I can communicate that some collaborators have announced themselves, and that the matter has been taken in hand on that account.

(*b.*) *The Calculation.*

1. The calculation is simple: the basis of it is the *measured decade-number.* It results from quietly counting 10 pressures with the finger upon the chronoscope and reading off the stand of the index; every watch-number is $\frac{4}{1000}$ seconds, and by division by 10, *i.e.*, striking off the last number by a comma, the *mean* number is obtained. Since the aggregate of the thus obtained numbers is too great for publication in the form of tables or curves, their number was diminished to $\frac{1}{4}$ by contracting every 4 to one *decade-mean.* This then, since the watch-number is 4 millseconds, gives immediately a number by simple addition, which expresses the obtained value in millseconds.

2. The *calculated decade-mean* is the base of a further calculation. This starts from the *water*-number (see above), and serves to determine how many per cents. the medicine-action had shifted the nerve-time in opposition to the time obtained at the water. Therefore, for every decade-mean obtained during the medicine-action, the difference of the water-number is taken and turned into *per cents.* of the water-number.

3. The *difference-numbers* in per cent. from the object of the demonstration in tables and curves and naturally are subdivided in two groups, with opposite signs. (*a*) The *minus*-sign was given to those numbers which indicate a *prolongation* of the nerve-time, therefore a *paralytic* effect. I call them *minus*-values; (*b*) the *plus*-sign is given to the numbers which indicate an *abbreviation* of the nerve-time, therefore indicate an *animating effect* and they are called plus-values.

4. For the formation of curves no further numbers were needed, but it was necessary to form a *single* sum-number which indicates the physiological value and renders a numerical estimation of each other possible. For this purpose the *plus* and *minus* values in the series of potency-numbers, which in the table are given for every potency, were added and from these by subtraction of the minus-values from the plus-values, and *vice versa*, the sum was found. This is done in the large table, No. 1, in the three last columns. Also the sum-numbers of course again are subdivided into *plus* and *minus*-values, *i. e.*, into numbers of animation and paralysis, and hence they received the corresponding signs.

(*c*.) *The Source of Error.*

Since we have to deal with physiological but not astronomical measurements, only two things are to be considered:

1. A fault of the chronoscope: after finishing the decade, the hand returns to its zero-position. In this operation it often happens that the hand remains a number from zero. Even if this would happen each time, which is not the case, this would for the decade-*mean* give only 0.4, and is therefore of no consequence whatever.

2. Of more importance is the physiological fluctuation, *i. e.*, how much do the decade-means obtained under equal proportions respectively at the equal object differ. Here is the question:

(*a*) Of the *rest*-number.—I have, as said before, taken measurements on four days. The following table gives the rest-numbers obtained each day in millseconds *seriatim* with the maximal difference each day and at the close, with the maximal difference from all 19 rest-numbers of the four days.

*Rest-Numbers.*

Jan. 19th. 92.7; 90.8; 91.0; 91.8; 90.3; 92.2. Maximal difference, 2.4.

Jan. 20th. 89.5; 91.1; 89.2; 90.0; 89.1; 89.6; 90.6. Maximal difference, 1.9.

Jan. 21st. 90.5; 91.9. Maximal difference, 1.4.

Jan. 25th. 91.9; 90.8; 90.2. Maximum difference, 1.4.

Maximal difference of all four days, 3.6

(*b*) Of the *water*-numbers which had been taken as the basis for the calculation of the medicine-action. The following table likewise throws light on this point, as the former, but not in absolute numbers, but by giving the difference in per cent. of the rest-numbers obtained previously.

*Water-Numbers.*

Jan. 19th. +1.4; —0.2; +1.1; +1.4; +0.3; —1.2; +3.4. Maximal difference, 4.6.

Jan. 20th. +0.5 ; +2.9 ; +1.2 ; +1.6 ; +0.4 ; +0.4 ; +2.5.
Maximal difference, 2.5.
Jan. 21st. —0.7 ; +1.1. Maximal difference, 1.8.
Jan. 25th. —1.3 ; —0.3. Maximal difference, 1.3.
Maximal difference of all four days, 4.7.

From this results for our medicine-numbers that differences of the decade-means of the table and of the curve, which amount to less than 5 per cent., are within the limit of error, but what amounts to more is of significance. Besides, since the measurements furnish differences which extend to the amount of 20 times of the possible quantity of error, the numbers cannot be questioned from this standpoint. Finally, since the physiological errors consist of opposite values, plus and minus, they cancel themselves mutually in the sum which is obtained by addition of all the values.

### (*d.*) *The Significance of the Numbers.*

The reader sees upon the great tables numbers of *three kinds :*

1. Those with *plus* signs : they signify, as already mentioned, an increase of velocity of the measured motion of life, are therefore the expression of a factor which accelerates the vital function, therefore develops " power " and acts the stronger, the greater is the number.

2. Those with *minus* signs : they signify a decrease of velocity, therefore are the expression of an influence which hinders, retards, paralyzes the vital motions, and acts the stronger, the greater is the number.

3. *Cyphers.*—They are only used when the difference in per cent. between water and medicine-number did not exceed the value of 0.5 ; they therefore signify *indifference.*

4. Among the numbers with plus signs those of 50 and higher are in bold face type. As soon as the animating action reaches or exceeds 50 per. cent, I observe upon myself *cramp*-symptoms, which later on will be considered extensively on account of their fundamental importance. Therefore call these numbers in bold face type in the following investigation *cramp*-numbers.

(*e.*) *The Table.*

There is little to be said about the *external* appearance.

1. The first column gives the number of the measured potency. The three last columns are explained above. The last is the most important, because it contains the numerical result of the *healing power* of the respective potency.

2. In regard to the upper horizontal line, with the statement of the *minutes*, the following must be said: The accurate measurement of the material power by Neural Analysis depends very much upon the prevalence of the utmost repose of mind during the measurement; neither the degree of concentration of attention nor its direction should be changed. Both would happen, if during the measurement of the decades one would simultaneously have to observe the second-hand of the watch and watch over their coincidence. Hence the marking of the minutes is the office of the assistant, and the measurer need not care about it. Therefore an exact division according to minutes *per se* is not feasible. To this must be added: The measurement generally furnishes in the minute 10 decade-numbers; since the decade-mean is formed from 4 decade-numbers, it does not exactly tally with one minute, but 5 numbers come upon 2 minutes. Therefore, numbers stand on the limits between the 1st and 2d, 3d and 4th, 5th and 6th minute. Twice, viz., in the 23d and 25th potency, it happened that in the space of time of 2 minutes 6 decade-means occurred, and the reverse with the 4 potencies, which took more than 7 minutes for measurement, the velocity of measurement slackened, so that only 4 decade-means came upon 2 minutes. This, of course, is not without influence upon the sum, but the result is not altered in the least, nay, the sum have a few points more or less.

3. From the table it is seen that the time of measurement of the various potencies was not equally long. I commenced at the lower potencies and continued to measure till I obtained several times consecutive numbers which coincided with the water-numbers, and I looked at this as a sign of the cessation of the action. This occurred in the 3d, 5th, and 7th potency at the

6th minute, in the 9th, 13th, 15th, at the end of the 5th minute. The 11th potency was the first which caused me to continue to measure. Since in general the return of the water-number under increase of the degree of potentiation showed a tendency to retardation I followed up the 25th and 27th in the 7th minute, the 30th in the 8th, and then continued to work the 50th for 10 minutes; this indeed had not been necessary, for the last mean-number, which is outside of the limit of error, is the first in the 8th minute; what then follows are 4 cyphers, once a +1 and a —1 cancelling themselves, and —3 being within the limit of error. The 100th potency was treated in the same manner and thereby the conviction (see the numbers) was gained that with the end of the 7th minute also here the action had ceased. Only then I went back again in order to solve the question whether in case of insufficient potentiation of the medicine which produced no animating effect, the organism perhaps afterward performed a potentiation. Then, especially in order to find out all about the favorite 6th potency, I took a second measurement of the 7th potency lasting 10 minutes, and this is incorporated in the table. As this second measurement (see the numbers) showed that I was right, when I, the first time, interrupted the measurement at the close of the 5th minute, for with a single very slight exception (+6) the 14 numbers of the 6th to 10th minute move within +3 and —1, are therefore within the limits of error.

4. In order not to make the table too broad, the measured tails in the 7th, 50th, 100th, and 1000th potency have been added in a second row.

### (*f.*) *Theoretical Preliminary Remark.*

In order to state the fact, that *much* matter, *heavy* matter, too—concentrated substances paralyze, saturate, retard the vital functions, and inversely small quantities, light, volatile, and attenuated substances animate, accelerate the vital motions, no Neural Analysis is needed; this is the experience of daily life and it is apparent. But a general question recommends itself.

The above statement *shows* that every substance disposes as it were of two *opposite* factors, a *retarding* or an *accelerating* factor. How can that be? What is the one and what the other? This must be clear before any explanation of the phenomena can be attempted.

The answer to this question is already in the sentences which enunciate the statement of the facts.

1. What is *paralyzing* or *retarding?* *Much* matter, *heavy* matter, concentrated substances. This immediately brings us to weight in the heavy substances, *e. g.*, metals, indirectly in regard to the much and too concentrated matter, because here the concept of the *mass* by which the weight is increased, intervenes. The factor can also be called *inertia*, for heavy substances have sluggish motions, likewise great masses, and the cencentrated substance is in want of space for motion.

2. What is *animating* and *accelerating?* Also here the answer is already in the words *light* and *volatile*. But here weight is not sufficient: one can indeed accelerate a motion mechanically by *diminishing* its weight, but never by *adding* to it another, ever so light weight. Under these circumstances a plus of the motion is by no means added to it. In order to accelerate a motion a *new* motion must be added to the existing one. In regard to this, however, it again is evident; if I shall add to a motion a *new* one of *less* velocity, therefore a *slower* motion, this signifies for the first one a loss, a *retardation*. If the velocities are *equal*, it remains as it was, nothing happens. Only when the newly-added motion has a *greater* velocity, it produces an *acceleration*. This brings me to the word *volatile*, to the fact that a substance is the better suited to produce acceleration of the vital motions the more volatile it is, and with this word we arrive at the factor "velocity of motion."

Now we are sufficiently prepared for the understanding of the fact which the irresistible logic of the numbers of our table preaches, and we can inasmuch as we desire it at all—for there are alas! people who do not want to understand in such matters—understand what happens in the potentiation of a medicine-substance. Whilst the quantity of the substance existing in the equal space (*e. g.*, the equal quantity of alcohol or the determined cubic contents of a living being) is diminished, the velocity of its *internal motion* is *increased*, not in quite equal but in similar manner (see my work on action of matter in living beings) as in *warming*, or it assumes the properties of a *volatile* substance, and these increase with the increasing attenuation, as the table clearly shows.

Hahnemann with perfect justice has called the process of attenuation "*Potentiation*," for it is indeed an *augmentation* of *force;* this is proved irrefutably by the physiological action, and it remains, now only to investigate, whether this force is the same which the physician calls the *healing power*, the *vis medicatrix*. Of this we shall speak further on, here we only want to add : if now in accordance with the modern molecular physics we call the phenomenon an increase of the velocity of the interstitial molecular motion, only this shall be said of it, that the phenomena observed and maintained by Hahnemann and thousands of his successors can be inserted perfectly and in the simplest manner in the scholarly frame of the physical theory and if then an extension of the limits of a city—I wanted to say of a conception cannot be avoided, this extension must even take place, for if a conflict between theory and fact occurs, the first must surrender unconditionally. I can throw the whole problem like a nut to be cracked at the physicist with a single word, the word *volatile*. But let us now pass from the theory to the fact; the theory will afterward at the hand of new facts, reappear again.

### (*g*.) *Examination of the Table.*

*Third potency*.—The first two numbers show the animating *first-action* sufficiently dilated upon in my former publications. It arises from the gradual diffusion of the substance entering the *primæ viæ* and from these into the whole circulation according to the laws of diffusion, and, of course, arrives in the nerves and muscles first in much lesser concentration, hence the first number is the highest, the second already smaller by one-half. The third shows already 25 per cent. paralysis, and thus it continues through the whole series, till at the close of the

### Table No. 1. Potencies of Kali-carbonicum.

| Potencies | 1. Minute 2 Minute | 3. Minute 4. Minute | 5. Minute 6. Minute | 7. Minute | Sum of Value | Sum of Value | Sum of both Values |
|---|---|---|---|---|---|---|---|
| 3 | +10 + 5 + 3 —25 —27 | —29 —30 —40 —30 —25 | —30 —18 —15 — 4 0 | | +18 | —273 | —255 |
| 5 | +20 + 3 + 4 —17 —21 | —19 —21 —32 —27 —36 | —37 —17 —21 —17 —8 | 0—5 0 | +27 | —278 | —251 |
| 7 | +15 — 8 —14 —24 —17 | —20 —21 —14 + 4 — 4 | + 3 — 2 + 2 — 1 + 2 | +6 0 +2 | | | |
| | | | 8. Minute 9. Minute | 10. Minute | | | |
| | | Forts. v. Pot. 7 | + 3 0 + 1 + 2 | +3+2 | +45 | —125 | —80 |
| 9 | +22 +38 +32 +17 — 7 | —24 —25 —23 —25 — 6 | +17 + 6 — 1 | | +132 | —111 | +21 |
| 11 | +17 +33 +39 +42 +44 | +39 +27 —11 —14 —15 | —17 —18 — 6 +23 +25 | +4+3 | +296 | —81 | +215 |
| 13 | +22 +58 +50 +42 0 | — 9 —10 + 6 +28 + 6 | 0 — 2 0 | | +212 | —21 | +191 |
| 15 | +20 + 9 +42 +62 +46 | +40 +18 + 8+ 14+ 42 | + 2 — 5 — 2 | | +303 | —7 | +296 |
| 17 | +48 +60 +66 +54 +58 | +54 +31 +20 +11 +15 | + 9 0 + 9 + 1 + 5 | | +441 | 0 | +441 |
| 19 | +57 +59 +62 +53 +55 | +55 +41 +44 +38 +31 | +30 +23 +15 +15 — 3 | —9 | +578 | —12 | +566 |
| 21 | +60 +71 +57 +43 +38 | +37 +54 +48 +22 +15 | +30 +29 +30 +20 — 1 | —5 | +554 | —6 | +548 |
| 23 | +62 +47 +77 +71 +78 | +75 +71 +61 +30 +14 +28 | +22 +21 +20 — 2 — 6 | | +677 | —8 | +669 |
| 25 | +70 +38 +30 +32 +40 | +48 +74 +78 +73 +72 | +45 +18 — 1 +22 +16 | —5—5 | +656 | —11 | +645 |
| 27 | +81 +83 +77 +76 +70 | +26 +33 +62 +46 +22 | +20 +17 + 9 0 —16 | —25—2 | +622 | —43 | +579 |
| 30 | +80 +78 +83 +77 +79 | +74 +49 +16 + 4 +25 | +63 +80 +79 +88 +76 | +50+9 —6 | | | |
| | | | | Forts. v. Pot. 30 —5 | +1010 | —11 | +999 |
| 50 | +71 +82 +82 +80 +76 | +70 +47 +41 +33 +25 | +42 +67 +83 +88 +77 +55 | +25+18 | | | |
| | | | 8. Minute 9. Minute | 10. Minute | | | |
| | | Forts. v. Pot. 50 | +14 0 0 0 + 1 | —32—1 | +1077 | —4 | +1073 |
| 100 | +84 +89 +90 +86 +83 | +85 +76 +64 +52 +35 | +27 +55 +83 +95 +98 | +74+30+10 | | | |
| | | | 8. Minute 9. Minute. | 10. Minute | | | |
| | | Forts. v. Pot. 100 | + 4 — 1 + 3 0 0 | 0 1 0 | +1223 | —2 | +1221 |
| 1000 | +91 +95 +93 +69 +68 | +80 +91 +92 +95 +90 | +78 +36 +31 +24 +32 | +32+28 | | | |
| Forts. v. | 8. Minute 9. Minute | 10. Minute 11. Minute | 12. Minute 13. Minute | 14. Minute | | | |
| 1000 | +28 +37 +26 +23 +22 | +24 +29 +25 +21 +24 | +26 +19 +19 +11 | +6 —2 | +1465 | —2 | +1463 |

sixth minute the action ceases. The sum —255 as a proof that the physiological action of this potency—the favorite third trituration—is nothing else than the paralyzing poisonous effect of the allopathic medicine-doses.

*Fifth potency.*—This is the same, but a slight amelioration is visible, (*a*) the numbers of the first action are better, their sum is greater; (*b*) the greatest paralytic number which with the 3d potency was —40, is here —32; (*c*) the sum, however, has gone back from —255 to only —251, which is still within the limit of error.

*Seventh potency.*—(*a*) Here the beginning is very instructive, since the *increase* of the *volatility* of the medicine shows itself in that the animating first action appears only in the first number; the substance has entered the blood and nerves quicker and thus the full action of this dose, a paralytic one, appears quicker; (*b*) the paralytic values are throughout lower, the maximum is with —21 by 11 lower than that of the 5th potency; (*c*) in the last case decided traces of an animating after-action appear; +3 is indeed within the limit of error, but +6 surpasses them within one and the same series of measurement; the physiological limits of error are much narrower than in two different series done at different times. Hence, I do not hesitate to consider also the plus-values 3, which occur three times, and the +2 as a sign that a medicine-action is there, and in this way: the body endeavors to restore the disturbed equilibrium to indifference by secretion of the medicine-substance, but in this effort it shoots beyond its aim. As the variation of small plus and minus values lasting through six minutes show this action struggles in vain for the mastery, and at any rate, nothing of value can be reached by it. Finally the sum of —80 condemns also this potency as insufficient, though it is only higher by one than the favorite 6th of the homœopathic domestic medicine-chests.

*Ninth potency.*—Again an increase which appears in the following: (*a*) a considerable animating first-action encompassing 4 numbers; (*b*) an undoubted animating after-action corresponding with the deconcentration by the secretory process. These advantages cannot be canceled by the circumstance that the middle phase of —25 lies 4 deeper than with the 7th potency, so that the sum now indicates by +21 that the Rubicon which must divide Allopathy and Homœopathy forever, viz.: *the point of indifference*, is passed—though hardly.

*Eleventh potency.*—This shows a more distinct increase than all the previous ones as already appears from the sums: the difference between the 3d and 4th potency is 44, that between the 5th and 7th is 131, that between 7th and 9th is 41, but that between 9th and 11th rises to 194 points. This increase of this potency, however, is owing only to a *one-sided* superiority, in reference to the *first action*, for this is higher and longer than that at the 9th potency, whilst the animating *after-action* is not much higher than that of the previous one. A further progress consists in the almost entirely lesser paralytic values of *the middle phase.*

*Thirteenth potency.*—This shows not only no increa-e, but even in the sum a decrease. Accident and error in measurement cannot be claimed for the explanation of this remarkable phenomenon, for it repeats itself from the 19th to 21st potency, from 23 to 25, and from 25 to 27. The occurrence, therefore, is owing to the nature of the case, and explains itself perhaps in this way: the *dynamic action* is always the product of *mass* and *velocity.* During potentiation evidently the *mass*, or, rather, in clinging to the molecular theory which we need not forsake for one moment, the *number* of the molecules decreases. If now, nevertheless, the motory effect does not decrease in the same amount, it proves quite incontrovertibly that the loss in mass is counteracted by the increase of the other factor, *i. e.*, of the *velocity.* It is thus not remarkable that degrees of attenuation occur in which the increase of velocity cannot quite replace the loss in mass or number and that the increase of power experiences a decrease or standstill. If we examine the series of numbers we see (*a*) increase in the appearance of the cramp-numbers in the phase of the first-action, and in the shortness of the middle phase with paralytic numbers and in the minuteness of these numbers; (*b*) the decrease is that the two periods of animation first and after-action last a very short while. This difference appears also in the two last columns; the sum of the paralytic values has gone down from 81 to 21; on the contrary, the animating values have not only not increased, but have gone down from 296 to 212.

*Fifteenth potency.*—This shows decided increase (*a*) in the first time, inasmuch as for the first time the minus-numbers in the middle phase are wanting, *i. e.*, the *middle phase* of the *paralysis* is wanting which separated the animating phase of the first and after-action in all the lower potencies. A decrease of action is there indeed and a very decided one, but it sinks no more below zero. (*b*) The after-action which in the 13th potency rose only to +28, here breaks through with +42 much livelier than hitherto, and on this subject I will speak a little more in detail, because it is very instructive for the comprehension of the medicinal-action. As in the pursuance of the work shall be proved by numbers, the intestinal canal has a regulatory influence, inasmuch as it protects the tissues from too rapid and great working of the materials introduced in it, and that in two ways, *i. e.*, toward the poisoning as well as to the animating exciting directions I should like to express it thus: the intestinal canal tries to down the material action, and in this it also succeeds up to a certain degree, but its power fails in two cases: once when the poisonous action of the first dose is sufficient to break the resistance of the intestines by *paralysis* and *then* also when the *volatility* of the substance has reached a certain height. And here a difference shows itself in regard to *first and after-action:* with the first-action the volatile materials rapidly supervene, and the intestinal canal succeeds in the suppression of the after-action. The last succeeds only when the volatility does not experience any further heightening. (*c*) Finally the 13th potency differs from the 15th, also by the longer duration of the first phase of animation and by the sum being greater by 105 than that of the 13th potency.

*Seventeenth potency.*—(*a*) This shows in the sum the significant increase of 145, (*b*) this is owing exclusively to the first-action, and less to the height which it attains (+66 to 62 of the 15th p.) than to the duration of the cramp-stage, which lasts five times longer. The 15th potency has only one cramp-number (the 17 5c). The after-action is very insignificant.

*Nineteenth potency.*—This shows (*a*) a considerable increase of power, from 441 to 556; (*b*) the peculiarity that the division in 3 phases, which we found to be regular even since the 7th potency, is here almost entirely wanting. It sets in at once with 5 cramp-numbers and then sinks, first gradually, later decidedly, and at last below zero.

*Twenty-first potency.*—Here we come a second time to a decrease in the sum, though only by 18 points. On proving the single values we find that this arises partly from the repetition of the middle phase with the lower numbers, so that again first and after-action can be discriminated (the last only very insignificantly), partly from the fact that it is one cramp-number less.

*Twenty-third potency.*—This shows again a considerable increase of the sum by 121 points. This arises from a considerable development of the first action, for it consists of no less than 8 cramp-numbers. The remission of action in the middle phase is marked by the number 14 distinctly, though weakly. The after-action of the third phase with the numbers 28—20 is distinct but still insignificant.

*Twenty-fifth potency.*—This shows a decrease by 24 points in its course so far a unicum as it is subdivided into five phases instead of three, since the first action is split by a phase of depression. To the high first number 70 follows a going down to 30, and this lasts into the third minute, in which only, with 4 energetic cramp-numbers, a lively action is indicated. Only in the beginning of the sixth minute the remission of the action ensues with —1, which corresponds with the middle phases of the other curves, and then a moderate after-action with 22+16 =38 points takes place.

*Twenty-seventh potency.*—Again, a decrease by 66 points and another likewise irregular picture behind in the region where otherwise the moderate after-action of animation takes place, a not inconsiderable abatement of the action with —16 and —25 ensues which has been marked already since the 19th potency. Its interpretation is perhaps best rendered by the word "fatigue" (from the previous cramps), otherwise the curve shows the ordinary picture: between two phases with cramp-numbers of which the first is longer than the second (in the 25 it was the reverse), a depression is expressed by the numbers 26 and 33.

*Thirtieth potency.*—This is the potency especially preferred by Hahnemann, and our proving is in perfect accordance with it. Already the extraordinarily high sum 999 appears like an event. The greatest increase of the sum in a difference of 2 potencies hitherto was 194, and should, with a difference of 3 potencies (27—30), amount to only 291 points, but we have a difference of 420 points. Here, therefore, we have the *greatest increase of the whole past series*, and this one circumstance must have been observed by Hahnemann. The second is the colossal likewise hitherto not existing development of the *after-action* which here occurs in the equal strength and duration as the *first action*. Every one consists of 6 cramp-numbers, and both are separated by a short but deep decrease of action going down to the number 4. The new experience therefore is that the after-action, which hitherto was very insignificant, breaks through victoriously. In the potencies from 17 to 25 it is so small that it could be overlooked by one who operated without Neural Analysis like Hahnemann. This was no more possible at the 30th potency, and had to make an impression of a new event: therefore, conformity of Hahnemann and Neural Analysis.

*Fiftieth potency.*—If we consider that between this and the 30th potency are 10 centesimal potencies, the increase of 74 points is very modest. It is also interesting that the mode of process coincides exactly with that of the 30th potency. The *same* equilibrium of first and after-action (only somewhat higher), and the same middle-phase of a remission (only likewise higher).

*Hundredth potency.*—The difference in the sum between the 30th potency with 999 and the 50th potency with 1,073, amounts to 20 decimal potencies $\frac{74}{20} = 3.7$; hence, every decimal potency increases the action of the medicine by 3.7 points. Now, the 50th and 100th potency differ by 50 potencies, and the sum of the 100th potency gives a plus of 148 points, therefore, a round increase of 3 points per potency; hence, the gain between 50 and 100 is less than between 30 and 50, but (for all that) worth a great deal. Further on the cramp-numbers reach into the 90's.

*Thousandth potency.*—Here friend Goullon cries: "What for?" to which I now answer, "for that," *i. e.*, to the following: the increase from 1,221 to 1,463, therefore by 242 points, has been laboriously reached under the production of 450 centesimal potentiation, which shows in the mean only a gain of half a point upon every centesimal potency, but it is not to be despised, and much less if we contemplate the series of numbers; for (*a*) this shows a highly important event: the action has for the first time a strikingly long tail. Hitherto the end of the action was reached in the beginning of the eighth minute, and here it continues into the 14th minute; this is a *Novum*. There is in it this circumstance worthy of observation, that here no cramp-attacks occur, but the after-action is a gentle and singularly equable one, expressed still more distinctly in the decade-number than in the decade-means of the tables. (*b*) Also, the *first* part of the series shows the power of this potency most clearly, because here without interruption 11 cramp-numbers, with the high total sum of 942 points occur, against 9 with 709 points in the 100th potency.

Herewith the reading of the first table is ended, and I hear the reader ask: "Why is it ended?" "Why not now the 2,000th, 3,000th potency?" To this I answer: in my first separately-published *Neural Analyse der Homœopathischen Verdengungen*, later in the third edition of *Entdeckung der Seele*, I have published my provings of attenuations beyond the 1,000th potency, and since I am firmly convinced that I would only find the same result, and that then I would have to say nothing else, as what I did before, I broke off with the 1,000th potency.

(*h.*) *The Curves.*

It contributes decidedly to the strengthening of the contemplation, if in all investigations which furnish numbers the same are used to make a *graphical* demonstration. This is done in the above, but with two limitations (*a*) of the 17 different potencies only 7 were demonstrated. In order to bring all 17 upon one net, a double size in every direction would have been necessary and even then an easy survey of the course of the lines would not have been possible without the application of different colors; (*b*) The curves give only every 17 numbers

### Neuro-analytic Curves for 7 different Potencies of Kali-carb.

First Minute | Second Minute | Third Minute | Fourth Minute | Fifth Minute | Sixth Minute |

of the table, therefore a space of time of somewhat over 6 minutes, for otherwise the drawing could not have been inserted in the frame of the text.

On the formality of the curves the following must be said : (*a*) the horizontal lines of the net determine the height of the measured number. The scale is marked along the perpendicular middle line and like a thermometer-scale indicates two values : *Above* the thick line of *indifference* marked *zero* the value receives the + sign (animating effects), below the minus sign (paralyzing effects). The numbers of the scale go from *ten* to *ten*, the lines from *twenty* to *twenty* because the drawing of more lines would have encroached upon the clearness of the drawing. Since the lines have 10 millimeter distances a point corresponds with half a millimeter.

(*b*) The *vertical* line marks the chronological sequence of the numbers of the table, and the table of curves has therefore the same superscriptions in minutes as the Table No. 1.

(*c*) The single curves are regulated by writing in the first column the potency which it is to represent ; then by the different drawing of the various lines by which they can easily be compared.

*Essentially* new things are not presented by the table ; it only makes it possible to see at a glance what in the other table must be read out of the numbers by slow degrees. I can, therefore, limit myself to the following :

*Fifth potency:* (punctated line) passes only in the first two columns over the line of indifference, and does not rise above it at the close.

*Eleventh potency:* (line of crosses) shows distinctly the three phrases, the first and last above, the middle below the line.

*Fifteenth potency:* (lower line in dashes) shows distinctly the chronological coincidence of the decrease of action in the 13th and 15th potencies, but also that the after-action in the 15th potency follows much more rapidly by heightening of the volatility and the decrease sinks no more below the line.

*Twenty-third potency:* (fine uninterrupted line) shows distinctly the long duration of the first action and that here the separation in a middle phase and a rising after-action is scarcely indicated.

The greatest simility is shown by the 30th potency (upper dash line) and by the 100th potency (thick uninterrupted line) in the distinct separation of the curve into two hills divided by a dale, which also coincide very nearly in their position with the only difference that in the 30th potency both hills are equal, in the 100th of unequal height and breadth.

*Thousandth potency:* (line of rings and points) shows the superiority of this potency over all others in the 4 first minutes of the action by its *strength.* Of the circumstance that the superiority depends upon the longer duration of the action whilst the strength is less, only the latter can of course be seen on the table.

[TO BE CONTINUED.]

## Figures and Tables

**Figure f1:**